# *Plasmodium vivax* associated severe malaria complications among children in some malaria endemic areas of Ethiopia

**DOI:** 10.1186/1471-2458-13-637

**Published:** 2013-07-08

**Authors:** Tsige Ketema, Ketema Bacha

**Affiliations:** 1 Department of Biology, College of Natural Sciences, Jimma University, P. O. Box 378, Jimma, Ethiopia

**Keywords:** Anemia, Hemoglobin, Parasitemia, *P. vivax*, Severe malaria

## Abstract

**Background:**

Although, *Plasmodium vivax* is a rare parasite in most parts of Africa, it has significant public health importance in Ethiopia. In some parts of the country, it is responsible for majority of malaria associated morbidity. Recently severe life threatening malaria syndromes, frequently associated to *P. falciparum*, has been reported from *P. vivax* mono-infections. This prompted designing of the current study to assess prevalence of severe malaria complications related to *P. vivax* malaria in Ethiopia.

**Methods:**

The study was conducted in two study sites, namely Kersa and Halaba Kulito districts, located in southwest and southern parts of Ethiopia, respectively. Children, aged ≤ 10 years, who visited the two health centers during the study period, were recruited to the study. Clinical and demographic characteristics such as age, sex, temperature, diarrhea, persistent vomiting, confusion, respiratory distress, hepatomegaly, splenomegaly, hemoglobinuria, and epitaxis were assessed for a total of 139 children diagnosed to have *P. vivax* mono-infection. Parasitological data were collected following standard procedures. Hemoglobin and glucose level were measured using portable hemocue instrument.

**Results:**

Median age of children was 4.25 ± 2.95 years. Geometric mean parasite count and mean hemoglobin level were 4254.89 parasite/μl and 11.55 g/dl, respectively. Higher prevalence rate of malaria and severe malaria complications were observed among children enrolled in Halaba district (P < 0.001). However, severe parasitemia was higher (72.4%) among children who visited Serbo health center (Kersa district). Male children had significantly higher risk of malaria infection (OR = 1.9, 95% CI, 1.08 to 3.34), while female had higher risk to anemia (OR = 1.91, 95% CI, 1.08 - 3.34). The observed number of anemic children was 43%, of which most of them were found in age range from 0–3 years. Furthermore, *P. vivax* malaria was a risk factor for incidence of anemia (P < 0.05) in the two sites.

**Conclusion:**

*P. vivax* associated severe malaria complications observed in this study was lower than those reported from other countries. However, incidence of severe malaria complications in one of the sites, Halaba district, where there is highest treatment failure to first line drug, could have significant impact on national malaria prevention and control activities.

## Background

*Plasmodium vivax* is the second important parasite of human malaria widely perceived as causing mild and self-limited illness. Unlike *P. falciparum*, it has wider geographical distribution. Though the public health importance of this parasite is overshadowed by *P. falciparum*, it is important parasite outside Africa, mainly in Asia and South America. It causes more than 390 million clinical cases per year [1]. *P. vivax,* previously thought benign parasite, is recently reported to cause life threatening complications among children from endemic regions such as Indonesia, India and Brazil [2-4]. Some of the reported severe malaria complications are cerebral malaria, dysfunction of different organs, hypoglycemia, jaundice, thrombocytopenia, renal impairment, hepatic dysfunction, acute kidney injury and hypotension [5-9].

*P. vivax* is a chief risk factor for severe anemia among young children in most *vivax*-endemic areas [2,10]. Though the mechanism of malaria associated severe anemia incidence is multi-factorial, there are established facts on intensive hemolysis of circulating infected RBCs, non infected erythrocytes due to glycosylphosphatidyl-inositol toxin released, and dyserythropoiesis that occur to the effect of different cytokines and other inducer of inflammation such as hemozoin [11-13].

Unlike other countries in Africa, prevalence of *P. vivax* infection in east Africa, particularly in Ethiopia is higher. In some areas of the country, the prevalence rate exceeds even 70% of total malaria infections. This was previously accounted to high Duffy blood group positivity of most population of the country [14], but recently contradictory reports are coming [15,16].

Although *P. vivax* malaria is highly prevalent in some parts of Ethiopia and its risk to drug resistance is increasing [17-20], to the authors’ knowledge study conducted on *P. vivax* associated severe malaria complication is almost none. Thus, it is rational to assess the current status of *P. vivax* related severe malaria complications in order to estimate the associated burden among biologically risked groups, children, in the endemic areas.

## Methods

### Description of the study area

**Figure 1:**
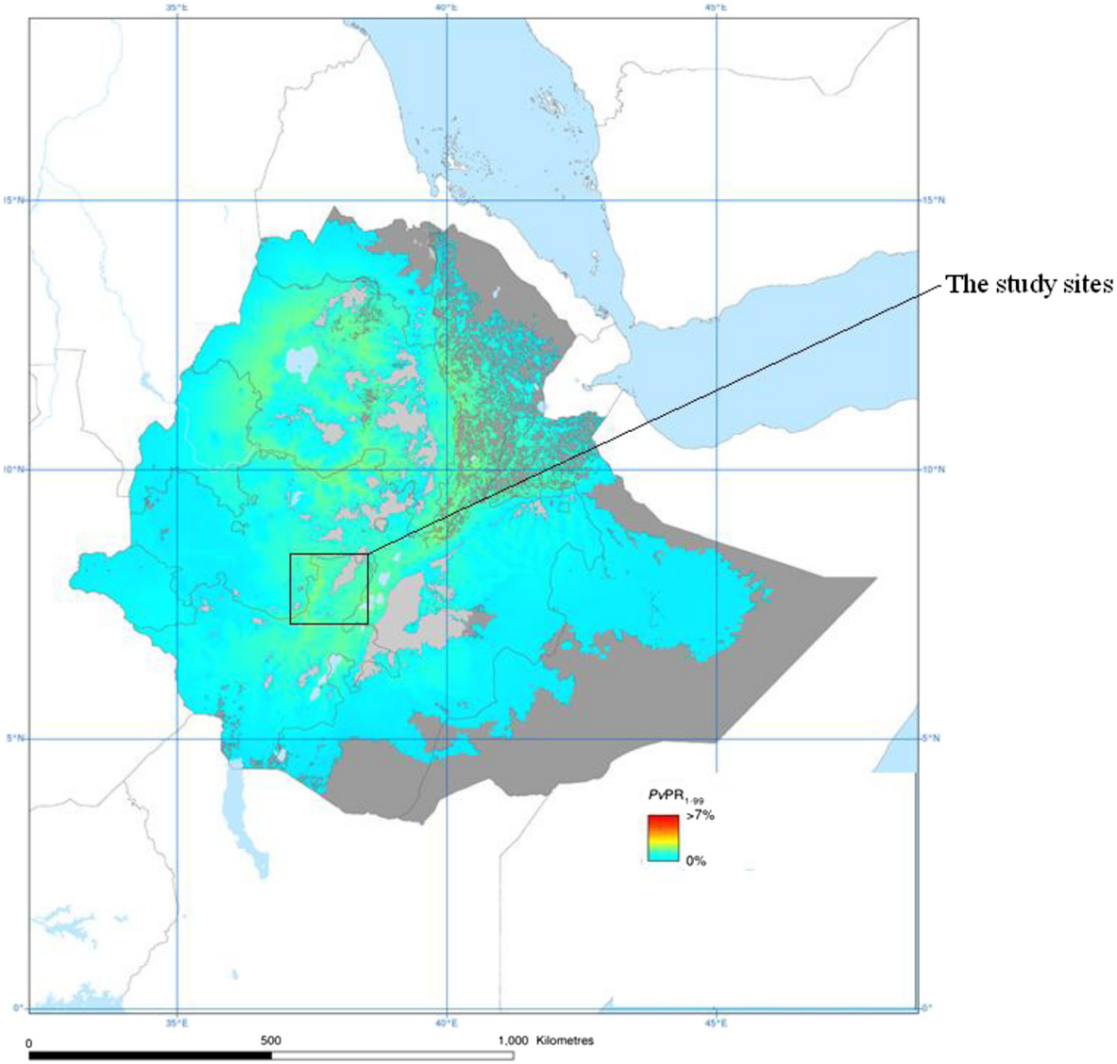
Map of the study sites.

The study was conducted in two of malaria endemic areas of Ethiopia, namely Kersa and Halaba districts, particularly at Serbo and Halaba Kulito health centers, respectively. The two sites were selected in order to compare incidence of severe *P. vivax* malaria complications in areas where there is intense *P. vivax* transmission (Halaba) and lesser transmission (Kersa). Geographically, Kersa district is located between altitudes ranging from 1,740- 2,660 meter above sea level (masl), which is relatively high land, where as Halaba district is found within altitudes of 1554–2149 masl (Figure 1).

The climatic zones of the districts have mainly consisted of mid-land and low-land. In Halaba, the most important parasite is *P. vivax* and it was responsible for more than 70% of the total malaria infection; whereas, *P. falciparum* is an important parasite in Kersa district and accounts for about 60% of all malaria infections in the area [19,20]. The mean annual rain fall is 1150 mm in Kersa and it ranges between 601–1200mm in Halaba. In addition, the average annual temperature ranges from 17.6- 22.5°C and 11.2 -29.6°C at Halaba and Kersa, respectively. In both areas, malaria is the most important health problem and the period from September to December is known as malaria peak season. The principal vector that transmits the disease in the country is *Anopheles arabiensis*.

### Study population

Study participants were children who visited the two health centers during the study period, September- December, 2009 and 2011, and had symptoms of malaria infection. The inclusion criteria used were: children aged ≤ 10 years, being infected with *P. vivax* mono-infection, without any chronic illness or not admitted to Tb and ART clinic, without prior medication, having clinical symptoms like fever, chills, malaise, headache, vomiting, history of fever for about 48 hours before admission, and volunteer to participate in the study.

### Data collection

Clinical and demographic data of the study participants (children) were recorded on pre-designed case record form by trained health professionals working at the two health centers. Accordingly, body temperature of each child was measured using digital thermometer; clinical symptoms such as fever, headache, hyperpyrexia, epistaxis, persistent vomiting, impaired consciousness, respiratory distress, hemoglobinuria, splenomegaly, and hepatomegaly were assessed. Children with at least one or more symptoms of severe malaria complications set by WHO [21] were classified as severe *P. vivax* cases.

Data from laboratory tests were collected by experienced laboratory technicians working in the health centers. Accordingly, a drop of blood sample was collected on clean glass slide from lancet pricked finger to prepare thin and thick blood smears in duplicate per patient for microscopic examination. Thick and thin blood smears were stained with 10% Giemsa (pH = 7.2, for 10 minutes), while thin smears were fixed in methanol prior to Giemsa staining. Malaria parasite was identified by observation of the smears and the morphological appearance of the parasite in the infected RBC under oil immersion objective. Parasite load was calculated after counting asexual parasites per 200 white blood cells (WBC), assuming mean WBC count is 8,000/μl. Each blood smear was examined by experienced laboratory technician in the health centers and then re-checked by certified laboratory technician at Jimma University. The degree of parasitaemia was graded as mild, moderate, and severe, when a count was between 1–999 parasite/μl, 1000-9999/μl, >10000/μl, respectively, following method described by Cheesbrough [22].

From the same pricked finger, few drops of blood samples were taken for measurement of hemoglobin (Hb) and blood glucose concentration (Glu) using handheld portable hemoglobin and glucose analyzer Hemocue™ (haemoglobinometer, Angelholm, Sweden). Hypoglycemia was considered when blood glucose concentration was < 40 mg/dl. Children with Hb level < 11 g/dl were considered anemic. Briefly, level of anemia was classified as severe, moderate and mild, when Hb concentration < 5 g/dl, between 5 and 8 g/dl, and between 8 and 11 g/dl, respectively, following the WHO anemia classification for severe *P. falciparum* malaria [21]. Hyperpyrexia was considered when body temp was > 40°C. All children were treated with chloroquine (25 mg base/kg) as per the recommendation of National Malaria Treatment Guideline [23].

### Data analysis

Data was analyzed using SPSS statistical software (version 16.0). Descriptive statistical tests were used for analysis of some clinical, demographic and parasitological data. Associations between variables were evaluated using Pearson correlation test and presented using matrix plot. Odd ratio was employed to describe strength of associations between variables in groups. Independent variables were compared using Mann–Whitney *U* test. Median was considered over mean for non-normally distributed variables. In all analysis, significance level was considered at 95% confidence interval.

### Ethical consideration

The study was ethically approved by Ethical Review Committee of College of Natural Sciences, Jimma University. Written consent/assent was obtained from guardians of the study participants prior to data collection.

## Results

### Malaria prevalence

**Figure 2:**
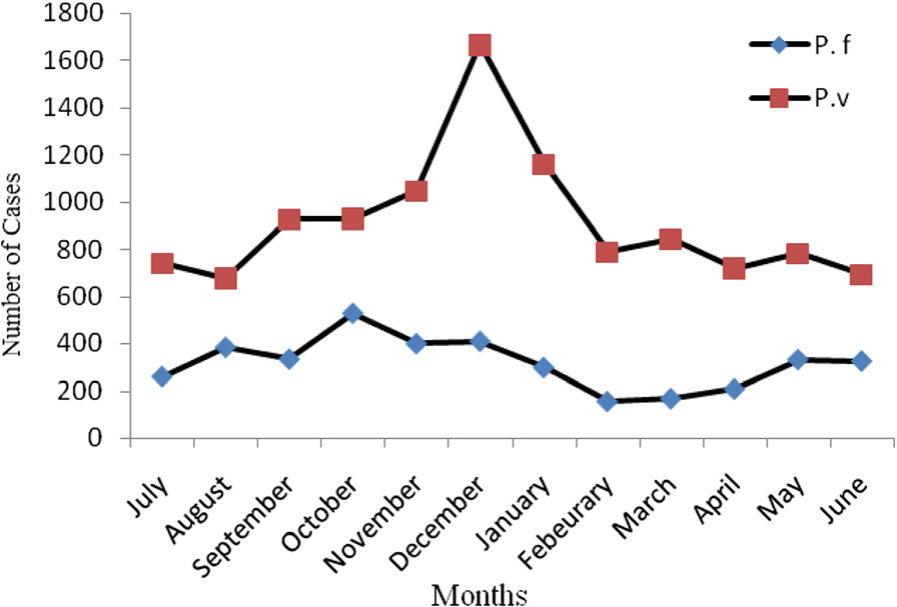
Number of malaria cases observed at Halaba district, SNNPR, Ethiopia.

During the study period, a total of 1497 blood samples were collected from presumptive malaria cases aged ≤ 10 years. About 31.9% (478/1497) were positive for malaria infection. Among these, 32.64% (156/478) and 67.36% (322/478) were infected with *P. vivax* and *P. falciparum,* respectively. Based on the inclusion criteria, a total of 139 children with *P.vivax* who fulfilled the inclusion criteria (n = 58 from Kersa and n = 81 from Halaba) were retrospectively analyzed. The rest (17/156) were excluded from the study due to prior medication, lack of consent from their guardians, and few due to admission to Tb clinic. The prevalence of *P. vivax* malaria was significantly different (P < 0.05) at Halaba district and significantly higher among children aged ≤ 5 years (P < 0.01). Sample composition in terms of sex and age did not differ significantly (p > 0.05) between the two study sites. Even if the overall malaria prevalence shows a declining trend across the country, the number of malaria patients is still high in Halaba district (Figure 2).

### Clinical and demographic characteristics

Based on the observed clinical and demographic characteristics, median age of the children was 4.25 ± 2.95 years. Male children had significantly higher risk of malaria infection (OR = 1.9, 95% CI, 1.08 to 3.34). At the time of admission, about 39% (54/139) and 21% (29/139) of the children had vomiting and diarrhea, respectively. Though most children, 94.2% (131/139) had a history of fever for the past 48 hours, only 55.4% (77/139) of them were febrile and had axillary temperature ≥ 37.5°C during enrollment. The median Hb and Glu concentration were 11.55 (4.7-15.2 g/dl) and 103 (36–170 mg/dl), respectively, while the geometric mean parasite count (asexual stage) and average body temperature were 4254.89 (320–36,590 parasite/μl) and 37.43 (36.5-40.7°C), respectively (Table 1).

**Table 1:** **Clinical and demographic characteristics of children infected with *P. vivax *at Halaba and Kersa districts, Ethiopia**

**S.No**	**Characteristics**	**Frequency (n = 139)**
1	Age (median)	4.25 ± 2.95 years
2	Sex	
	• Male	58% (n = 80)
	• Female	42% (n = 59)
3	Fever at time of enrollment	55.4% (n = 77)
4	History of fever for about 48 hours	94.2% (n = 131)
5	Mean body Temp (range)	37.43 (36.5-40.7°C)
6	Vomiting	39% (n = 54)
7	Diarrhea	21% (n = 29)
8	Average days of illness (range)	2.92 (2-7 days)
9	Anemic cases (Hb < 11 g/dl)	43% (n = 60)
10	Median Hb level (range)	11.55 (4.7-15.2 g/dl)
11	Median Glucose concentration (range)	103 (36–170 mg/dl)
12	Geometric mean parasite (range)	4254.89 ±8104.3
		(320–36,590 parasite/μl)

**Figure 3:**
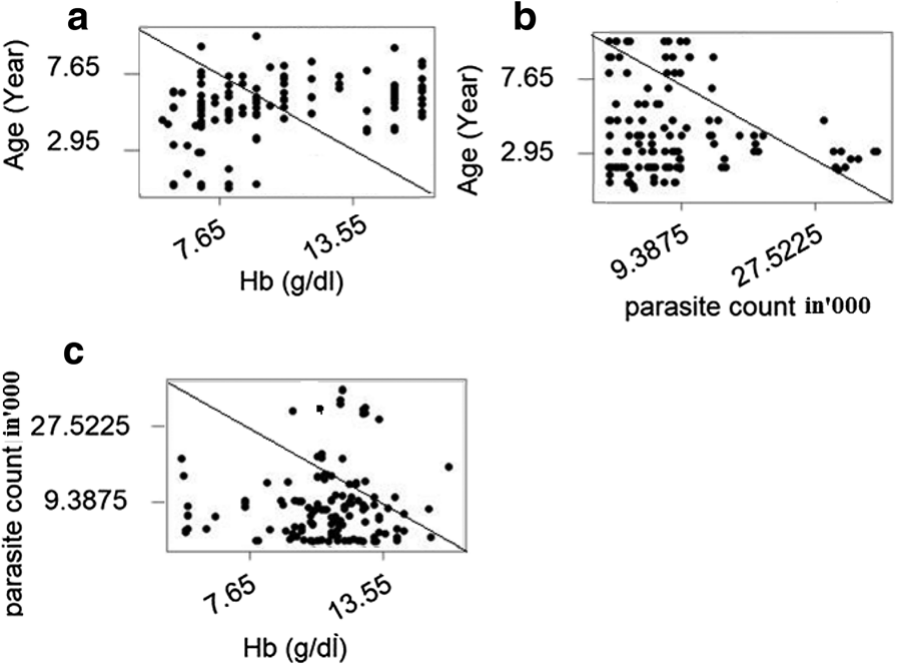
**Association between age, hemoglobin level and parasite counts among children infected with *P. vivax *at Halaba and Kersa districts, Ethiopia.**

Pearson correlation analysis revealed that age of children was negatively correlated to parasite count (r = −0.2358, p < 0.05) but had positive correlation to hemoglobin levels. Accordingly, as age of children increased the parasite count decreased, while the level of Hb increased (r = 0.31, P < 0.001). However, there was no significant differences (r = 0.057, p > 0.05) between Hb level and parasite count (Figure 3).

### Severe malaria

Among 139 children, a total of 19 (13.67%) fulfilled at least one of the WHO criteria for severe malaria [21]. Some of these syndromes were; persistent vomiting (10.5%, n = 2), respiratory distress (15.8%, n= 3), hypoglycemia (10.5%, n=2), hyperpyrexia or temperature >40°C (21%, n = 4), and severe anemia (42%, n = 8). But, none had symptoms like hepatomegaly, splenomegaly, epistaxis, confusion or coma, and hemoglobinuria or discoloration of urine. Severe malaria syndromes were significantly higher (OR = 3.16, 95% CI, 1.77- 5.6) among children who visited Halaba Kulito health center (Table 2). Children with severe parasitemia, parasite count >10,000 parasite/μl, were significantly higher (P < 0.001) in Kersa (Serbo health center). Likewise, vomiting cases were significantly higher (OR = 2.52, 95% CI = 1.39 - 4.57) among children enrolled at Serbo health centre. However, incidence of diarrhea and fever were not significantly different (P > 0.05) between the two sites. Number of children with more than one severe malaria symptoms were very few (n = 6) and most of them had severe anemia with other syndromes including persistent vomiting (n = 1), respiratory distress (n = 3), and hyperpyrexia (n = 2).

**Table 2:** **Prevalence of severe malaria complications among *P. vivax *infected children at Halaba and Kersa districts, Ethiopia**

**S. No**	**Characters**	** Sites**	
		**Halaba**	**Kersa**
1	Malaria prevalence	n = 81 (58.27%)	n = 58 (41.72%)
2	Anemic children (Hb < 11 g/dl)	n = 37 (45.68%)	n = 23 (39.65%)
3	Severe anemia (Hb < 5 g/dl)	6	2
4	Hypoglycemia (Glu < 40 mg/dl)	2	0
5	Severe parasitemia (>10,000 Para/μl)	8	21
6	Respiratory distress	2	1
7	Hyperpyrexia	1	3
8	Persistent vomiting	1	1
9	Vomiting	21/81(25.9%)	27/58 (46.55%)
10	Diarrhea	23/81 (28.39%)	14/58 (24.1%)
11	Febrile	48/81 (59.26%)	29/58 (50%)

The frequency of incidence of anemia (43%, ± 9.7, 33.3-53%) was significantly higher (P < 0.05) among children within the age range of 0 - 3 years than other categories (Table 3). Severe anemia was observed in eight children aged between 0–3 years (n = 7) and 4 years (n = 1). Most severe anemic cases (75%, n = 6/8) were observed in Halaba district. In terms of sex, female children had significantly higher risk of anemia (OR = 1.91, 95% CI, 1.08 - 3.34). Severe parasitemia was significantly different (OR = 0.1854, 95%CI, 0.088 - 0.39) among anemic children. The study showed that *P. vivax* malaria was a risk factor for incidence of anemia (P < 0.05).

**Table 3:** **Incidence of severe anemia, parasitemia and hypoglycemia among children infected with *P. vivax *at Halaba and Kersa districts, Ethiopia**

**No**	**Age (year)**	**Hb level (g/dl)**			**Parasite count/μl**			**Glucose level (mg/dl)**	
		**< 5**	**5 < Hb < 8**	**8 < Hb < 11**	**1-999**	**1000-9999**	**>10000**	**< 40**	**>40**
1	0-3	7	11	15	7	28	18	2	35
2	>3-6	1	5	10	11	22	13	0	33
3	>6-10	0	0	11	15	15	10	0	30
Total		8	16	36	33	65	41	2	137

Severe parasitemia was observed in 41 children with almost similar proportion in all age groups except for children < 3 years (43.9%, n = 18/41) who was found to have higher parasitemia. Hb level and parasite count of children in the age category of 0–3 years was compared with those in the highest age category (>6 years) using Mann–Whitney *U* test. The result showed that Hb level was significantly different (P < 0.01) between the two age categories, but parasite count didn’t show significant difference (P > 0.05). Parasite counts of all children with severe anemia were found in the range of 1000–9999 parasite/μl.

## Discussion

The prevalence rate of malaria observed in this study was 31.9%, which was much lower than the very recent report from Nigeria (81.9%) [24]. This might be due to the intense and diverse malaria control strategies undertaken in most parts of Ethiopia. The intervention made so far has significantly reduced prevalence of malaria in some endemic areas. As evidence, during the second phase of this study (in 2011), the numbers of malaria patients observed at Serbo health center were very few for recruitment in the study. At Halaba district, however, though the trend of malaria prevalence appears declining, the numbers of malaria patients visiting the health center were still high. For instance, in the same year (2011), among a total of 27,499 malaria suspected patients, 14,775 (53.73%) were found positive. Out of these, 10,957 (39.8%) were due to *P. vivax* and the rest were accounted to *P. falciparum* (Unpublished data, Annual Report on Malaria Prevalence by Halaba Kulito health center, 2011).

Most of the children enrolled in this study were found in age group ≤ 5 years. In this age category higher load of parasitemia and incidence of severe anemia, but lower concentration of hemoglobin was observed. This strengthens the fact that children in this age group who live in holo or hyperendemic areas are biologically risked group [25]. This is because of the development of poor immunity against the disease [21], but as they get older and repeatedly exposed to the disease they gradually develop protective immunity to malaria [26].

Percentage of anemic cases documented in this study (43%) was higher than the earlier 34% reported from children [27] and 30.5% from among adults [28] in South–Western India. Usually anemia is associated with intense hemolysis of RBCs due to higher parasitemia such as in the case of infection with *P. falciparium*. However, due to selective preference to only young RBCs by *P.vivax*, it appears that the number of hemolyzed RBC during *P.vivax* is minimal. Thus, the incidence of anemia might occur as a result of rigor inflammatory reactions due to pro-inflammatory response and cytokines activation [29] and less deformability of RBCs during *P. vivax* infection [30,31]. On the other hand, the rate of non infected RBCs hemolysis for every infected RBC destroyed could contribute to the incidence of anemia as number of non-parasitized RBCs removed from circulation during *P. vivax* is much higher (~32) than *P.falciparum* (~8) [32,33].

Frequency of *P.vivax* associated severe malaria complications documented in this study (13.67%) was less than those report from Eastern Sudan (18%) from among children admitted to hospital due to severe *P. vivax* malaria [8], but slightly higher than those from Venezuela (10.26%) [34]. In agreement with report from Eastern Sudan [8], the most prevalent severe malaria complication observed in this study was severe anemia (36.36%) followed by hyperpyrexia, respiratory distress and persistent vomiting. Incidence of hypoglycemia was minimal. Other severe malaria symptoms including hepatomegaly, splenomegaly, confusion, epistaxis and persistent vomiting were not observed, except the respiratory distress cases which was in agreement with the recent report from India [35].

With regard to sites, the numbers of children with severe malaria complications were higher among those children who visited Halaba Kulito health center, the site where the highest (13%) treatment failure to chloroquine (the first line drug) by *P.vivax* malaria was documented [20]. Siliva-Filho et al. [36] elaborated that when there is drug resistance trait in a certain area, it increases peripheral parasitemia for a longer time and enhance hemolysis of RBCs. As a consequence, persistent of the parasite in hosts blood can increase incidence of severe malaria syndromes. This is evidenced by frequent occurrence of severe anemia in such localities [37]. On the other hand, intense disease transmission in Halaba district might have contributed to the higher severe malaria cases. It is also affirmed that in an area where there is intense malaria transmission there is high incidence of severe malaria morbidity which results in higher hospital admission among young children [38].

## Conclusion

As *P.vivax* malaria parasite had already developed resistant to the first line drug, chloroquine, in one of these study sites, underestimation of complications caused by *P. vivax* malaria could have significant impact on current malaria control and prevention activities being under taken in the districts. Thus, it calls for special attention and frequent surveillance by concerned bodies in order to achieve the sought national malaria reduction plan.

## Competing interest

The authors declare that they have no competing interest.

## Authors’ contributions

Both authors equally involved in all phases of the study including designing of the study, data collection and monitoring, data analysis, and write-up of the manuscript. Both authors read and approved the final manuscript.

## Pre-publication history

The pre-publication history for this paper can be accessed here:

http://www.biomedcentral.com/1471-2458/13/637/prepub
